# Fano Resonance in Waveguide Coupled Surface Exciton Polaritons: Theory and Application in Biosensor

**DOI:** 10.3390/s18124437

**Published:** 2018-12-14

**Authors:** Jiaqi Zhu, Shuaiwen Gan, Banxian Ruan, Leiming Wu, Houzhi Cai, Xiaoyu Dai, Yuanjiang Xiang

**Affiliations:** College of Optoelectronic Engineering, Shenzhen University, Shenzhen 518060, China; 2161190233@email.szu.edu.cn (J.Z.); 2172281559@email.szu.edu.cn (S.G.); 2161190229@email.szu.edu.cn (B.R.); wlm40588@163.com (L.W.); xiaoyudai@126.com (X.D.); xiangyuanjiang@126.com (Y.X.)

**Keywords:** surface exciton polaritons, J-aggregate cyanine dye, fano resonance

## Abstract

Surface exciton polaritons (SEPs) are one of the three major elementary excitations: Phonons, plasmons and excitons. They propagate along the interface of the crystal and dielectric medium. Surface exciton polaritons hold a significant position in the aspect of novel sensor and optical devices. In this article, we have realized a sharp Fano resonance (FR) by coupling the planar waveguide mode (WGM) and SEP mode with Cytop (perfluoro (1-butenyl vinyl ether)) and J-aggregate cyanine dye. After analyzing the coupling mechanism and the localized field enhancement, we then applied our structure to the imaging biosensor. It was shown that the maximum imaging sensitivity of this sensor could be as high as 5858 RIU^−1^, which is more than three times as much as classical FR based on metal. A biosensor with ultra-high sensitivity, simple manufacturing technique and lower cost with J-aggregate cyanine dye provides us with the most appropriate substitute for the surface plasmon resonance sensors with the noble metals and paves the way for applications in new sensing technology and biological studies.

## 1. Introduction

In the past several years, research on polariton has drawn great attention due to the mature development between experiment and theory. It is easy to understand the concept of polariton, which is the strong coupling between a photon and another quasiparticle. It is well known that there are three main types of polariton: Plasmon polariton, phonon polariton, and exciton polariton [1]. The plasmon polaritons have been studied extensively in visible wavelengths, and the phonon polaritons are widely researched in the infrared region [2,3,4,5,6], but the novel biosensors are seldom researched based on exciton polaritons. In 2017, Kentaro Takatori et al. proposed novel surface exciton polariton (SEP) biosensors based on a Kretschmann-Raether device, and the researcher experimentally validated that SEPs propagate along the interface of J-aggregate cyanine dye-air in ambient conditions. Its most notable feature is that J-aggregate cyanine dye is a simple fabrication technique, and, from its polar solution, it can be simply deposited on the substrate by means of spin-coating and dip-coating at room temperature [7]. In this paper, we use the dye 5,5′,6,6′-tetrachloro-1,10-diethyl-3, 30-di(4-sulfobutyl) benzimidazolocarbocyanine (TDBC) as the cyanine dye, which can be easily deposited on the substrate by dip-coating or spin-coating, though it is partially crystalline. As regards TDBC, much research has been done on the strong coupling between TDBC and other modes [8,9,10]. Compared with traditional noble metals like gold, J-aggregate cyanine dye has distinct advantages in production cost and fabrication techniques, because the cost of the gold chip is more than ten times that of cyanine. On the other hand, the chemistry property of organic crystals is more stable than precious metal. Therefore, SEPs may realize a simple and low-cost biosensor in ambient condition and mass production.

In order to obtain better performance of nanostructure sensors, researchers target coupling for different electromagnetic modes. One approach is to employ Fano resonance (FR), which is of growing interest and widely used in sensing [11,12]. According to plasmon resonance mode, FR normally arises from hybridization (interference) of bright (radiation) mode and dark (sub-radiant) mode. The bright mode originates from dipole oscillations and has wide spectrum characteristics due to radiation damping properties. On the contrary, the dipole moment of the dark mode is almost zero, which cannot effectively couple with incident light waves. The two neighboring resonators have a significant effect on optical behavior due to the near-field coupling effect among them, which leads to a localized electromagnetic field distribution in the sub-radiant resonator [13]. It has come to our knowledge that FR is realized by coupling two different resonances (a broad and narrow resonance), and the greatest distinctive features are sharp asymmetric spectral line shape, rapid changes in the aspect of phase [14] and amplitude [15]. In recent years, FR has been widely used in most applications such as single molecule detection, Goos–Hänchen shift, surface-enhanced spectroscopy (SES), high-sensitivity sensors [16,17,18,19,20] and so on.

In this article, we propose a multilayer thin film biosensor based on FR, and, as far as we know, the coupling between SEP mode and waveguide mode (WGM) has not been studied so far in the visible spectrum. Therefore, we design an ultrasensitive biosensor by realizing the coupling between WGM and SEP mode. The sharp asymmetric curve of FR is shown in the angular spectrum, after numerical calculation, and we also prove that the proposed biosensor sensitivity has at least tripled in intensity compared to traditional FR based on noble metal [21].

## 2. Design Consideration and Theoretical Model

**Figure 1:**
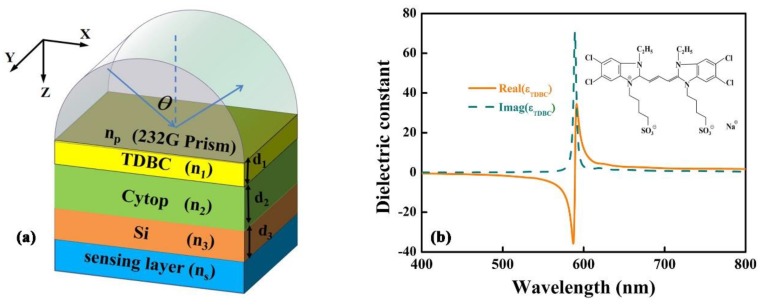
(**a**) The schematic view of the designing biosensor. (**b**) The complex dielectric function of the TDBC film.

Figure 1a shows the diagrammatic drawing of the proposed structure. In this configuration, TDBC layer is attached to the chalcogenide glass (2S2G) prism, we choose silicon (Si) as the waveguide layer and the cladding layer between WGM and SEP mode as Cytop. Like conventional SPR sensor, the proposed structure Prism-TDBC-dielectric can form a SEP sensor due to SEP modes propagated along the boundary between TDBC and dielectric. In this article, the structure Cytop-Si-Sensing medium can support WGM, due to the fact that the waveguide layer Si is surrounded by Cytop and sensing medium and the index of refraction larger than them; the structure of waveguide mode is referred to in reference [22], which presents experimental and theoretical research about the excitation of long-range surface polaritons based on α-Si. We believe that it will be possible to realize the coupling of the two modes of SEP and waveguide only if we choose the appropriate materials and the thickness of the coupling layer. Figure 1b is the calculated permittivity of the TDBC film: we can see that the TDBC surface excites SEPs ranging from 463 nm to 589 nm, and the inset figure is the chemical structure of TDBC.

We choose the 2S2G prism as the coupling layer, and its index of refraction follows the relation [23]:

(1) $$n_{p}=\;2.24047\;+\;\frac{2.693\;\times \;10^{-2}}{\lambda^{2}}\;+\;\frac{8.08\;\times \;10^{-3}}{\lambda^{4}},$$

where *λ* is the wavelength of incident light in micrometers.

In this calculation, the permittivity of the TDBC film is shown in reference [7], and the parameters are shown as follows:

(2) $$\varepsilon_{1}\left(\omega \right)=\varepsilon_{\infty }+\sum_{j=1}^{5}\frac{\omega_{pj}^{2}}{\omega_{0j}^{2}-\omega^{2}-i\gamma_{j}\omega },$$

where the unit is cm^−1^ and fitting paraments are *ω_pj_* (*j* = 1, 2, 3, 4, 5) = 4340, 4383, 3511, 11,830 and 1621, *ω*_0*j*_ (*j* = 1, 2, 3, 4, 5) = 13,570, 15,330, 16,140, 16,960 and 18,710, *γ_j_* (*j* = 1, 2, 3, 4, 5) = 2409, 1352, 565.5, 117.3 and 561.6, and *n*_1_ = *ε*_1_^1/2^.

We choose Cytop as the coupling layer and the corresponding refractive index *n*_2_ = 1.34. We choose the Si as the waveguide layer; its complex refractive index is given as the relation [24]:

(3) $$n_{3}=A+A_{1}e^{-\lambda /t_{1}}+A_{2}e^{-\lambda /t_{2}},$$

where *A* = 3.44904, *A*_1_ = 2271.88813, *A*_2_ = 3.39538, *t*_1_ = 0.058304, *t*_2_ = 0.30384.

The sensing medium for initial calibration is deionized (DI) water and its refractive index (*n_s_*) is determined by the following relation [25]:

(4) $$n_{s}^{2}-1=\sum_{i=1}^{4}\frac{A_{i}\lambda^{2}}{\lambda^{2}-t_{i}^{2}},$$

where *A*_1_ = 5.666959820 × 10^−1^, *A*_2_ = 1.731900098 × 10^−1^, *A*_3_ = 2.095951857 × 10^−2^, *A*_4_ = 1.125228406 × 10^−1^, *t*_1_ = 5.084151894 × 10^−3^, *t*_2_ = 1.818488474 × 10^−2^, *t*_3_ = 2.625439472 × 10^−2^, *t*_4_ = 1.073842352 × 10^−1^, and *λ* is the wavelength of incident light in micrometers.

In this configuration, the classic dispersion relation of two semi-infinite dielectric layers to calculate the SEP may not match the numerical. Then we choose the prism, TDBC and cytop to compose a three-layer structure. By meeting the boundary conditions for the *n_p_*-*n*_1_-*n*_2_ system, we calculate the SEP dispersion as follows:

(5) $$\tanh(\alpha_{1}d_{1})=-\frac{\Gamma_{p}+\Gamma_{2}}{1+\Gamma_{2}\Gamma_{p}},$$

where Γ*_p_* = (*ε*_1_*α_p_*)/(*ε_p_α*_1_), Γ_2_ = (*ε*_1_*α*_2_)/(*ε*_2_*α*_1_), $a_{j}=\sqrt{\beta^{2}-k_{0}^{2}\varepsilon_{j}}$, *j* = *p*, 1, 2, *β* is the *x* component wavenumber, *ε_j_* is the corresponding permittivity, and *k*_0_ = *ω*/*c*_0_ is the free space wavenumber.

As for the three-layer waveguide structure (Cytop-Si-Sensing medium), the dispersion of the planar waveguide (PWG) mode can be derived as:

(6) $$\tan k_{3z}d_{3}=\frac{k_{3z}\left(p_{2}\alpha_{2}+p_{s}\alpha_{s}\right)}{k_{3z}^{2}-p_{2}\alpha_{2}p_{s}\alpha_{s}},$$

where *p_i_* = *ε*_3_/*ε_i_*, $k_{3z}=\sqrt{k_{0}^{2}\varepsilon_{3}-\beta^{2}}$ and *i* = *s*, 2.

To calculate the reflectivity change of the proposed multilayer configuration, we can obtain the angular spectrum after calculation and theoretical modeling from Fresnel equations and the transfer matrix method (TMM) [26,27], which is a function of the angle of incidence θ*_in_*. Under the condition of the incident TM-polarized light, we use the TMM to analyze the reflectance, and the sensitivity is defined as *S* = *dR_p_*/*dn_s_* [28]. The TM-polarized light is 532 nm in the proposed structure and is assumed to be incident light.

## 3. Results and Discussion

**Figure 2:**
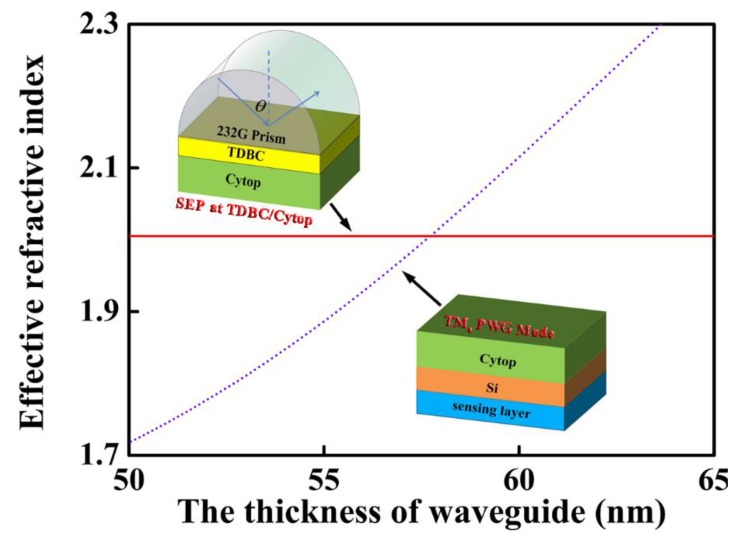
Dispersion relations of surface exciton polariton (SEP) mode (red solid line) and waveguide mode (WGM) (violet dotted line).

First of all, we must check the coupling condition of the SEP mode and WGM in advance in order that we compute the numerical calculations of reflectivity. Here, we should separately consider the SEP mode and WGM: The SEP mode propagates along the boundary of TDBC-Cytop, and the WGM is supported by a waveguide surrounded by Cytop and water (sensing medium), as shown in inset picture of Figure 2. We can easily solve the dispersion relation of Equations (5) and (6) by the numerical method and obtain the effective index by the relational expression of *n_eff_ = β*/*k*_0_. When the two modes of SEP and waveguide are matched, they can couple together and excite FR. In Figure 2, we can clearly see that the effective refractive indexes between SEP mode and WGM vary in terms of the thickness of waveguide (d_3_), and it is found that the curve of SEP mode is a straight line because it is independent of the waveguide layer (Si) and can be altered by geometric structure and incident light wavelength. As the thickness of the waveguide layer increases, the curves of PWG mode and SEP mode intersect at one point around d_3_ = 58 nm. Around the crossing point, two modes can be excited simultaneously, and hence the mode coupling comes into being. 

**Figure 3:**
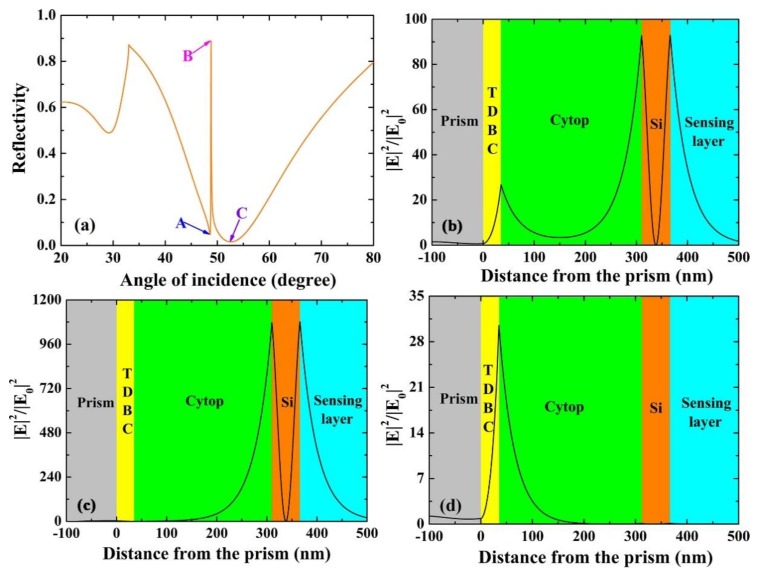
(**a**) The angular reflection spectra calculated as a function of the angle of incidence with d_1_ = 35 nm, d_2_ = 300 nm, and d_3_ = 56 nm. (**b**–**d**) present an electric field (|E_X_|^2^/|E_0_|^2^) corresponding to the three dips denoted as “A”, “B” and “C” in Figure 3a, respectively.

The angular spectrum of the proposed multilayer structure is easily derived, as shown in Figure 3a; there, only zero order is propagating and no other modes. It can be seen from the diagram that both SEP mode and WGM can be excited: A broad resonance dip appears which indicates the excitation of SEP modes in θ = 52.6°, and a narrower reflection dip appears which indicates the excitation of WGM in θ = 48.81°. The most distinctive feature of FR is a sharp asymmetric line shape in the reflection spectrum. It is necessary for us to illustrate the origin of sharp resonances, and we calculated amplitude distribution of the electric field respectively (corresponding to the three dips denoted as “A”, “B” and “C”). We know that the electric field appears in a certain region with distinct differences according to the distribution profile. As shown in Figure 3b, corresponding to point “A”, we see that the electric field strength primarily distributes within TDBC and Si layer, which is generated by incomplete excitation of SEP mode and WGM. We require the coupling between SEP mode and WGM to take place in the waveguide layer with a high electric field, as shown in Figure 3c for the point “B”, where the electric field enhancement factor (|E|^2^/|E_0_|^2^) is defined as the ratio of the square of the electric field amplitude to that of incidence [21]. We should pay attention to the fact that an enhancement factor as high as 1076 at the interface of the Si-sensing medium has been obtained. In Figure 3d, we see that the electric field mostly appears around TDBC layer and decays exponentially away from the interface of TDBC-cytop, which is the specific feature of the excitation of SEP mode corresponding to point “C” in Figure 3a.

**Figure 4:**
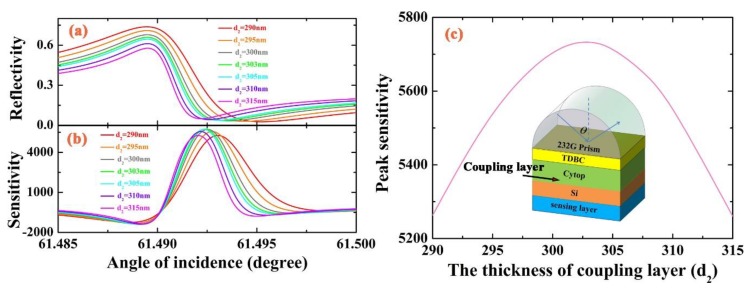
(**a**–**c**) Variation of reflection spectra, sensitivity and peak sensitivity with respect to the thickness of coupling layer (Cytop) d_2_ ranging from 290 to 315 nm, where λ = 532 nm, d_1_ = 35 nm, d_3_ = 62 nm and *n_s_* = 1.327.

After making it clear that the two modes of SEP and WGM can be coupled together and finding out the origin of sharp Fano line shape, we should further discuss and optimize the proposed structure. We know that the strength of coupling between the SEP mode and WGM is regulated by the overlap of their evanescent fields, hence the thickness of the coupling layer d_2_ to control the coupling strength. It is obvious that the coupling strength is weaker when d_2_ is very thick. As we gradually increase the distance between the TDBC and Si layer (coupling layer Cytop), a narrower resonance appears, due to the decrease of intrinsic and radiation loss of WGM [29]. However, Figure 4a shows that the resonance becomes sharper and then degrades when we gradually increase the coupling layer d_2_, which means a decrease in coupling strength. Figure 4b shows the corresponding sensitivity. In order to clearly show the influence of the thickness of the coupling layer on sensitivity, we plot Figure 4c to determine the optimal thickness of Cytop. We can obtain the maximum sensitivity S = 5739 RIU^−1^ with the thickness of Cytop d_2_ = 303 nm from the peak sensitivity figure. As a result, we can see that the sensitivity first goes up to a certain value, then decreases. Considering practical application, we choose the thickness of Cytop d_2_ as 300 nm.

**Figure 5:**
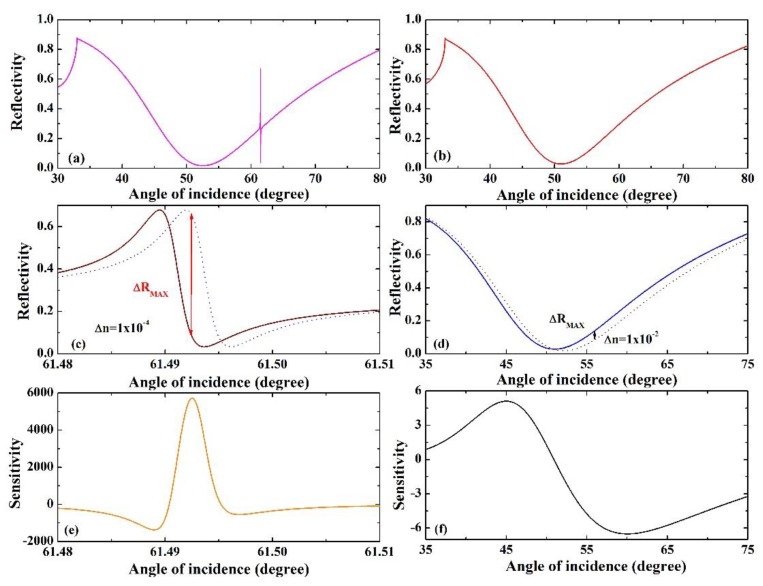
(**a**) Sharp resonance arising from the coupling between SEP mode and WGM for λ = 532 nm, d_1_ = 35 nm, d_2_ = 300 nm, d_3_ = 62 nm and *n_s_* = 1.327. (**b**) Traditional SEP sensor for λ = 532 nm, d_1_ = 35 nm and *n_s_* = 1.327. (**c**,**d**) The shift of the Fano-type resonance for the different coupling mode and conventional SEP sensor, caused by the different refractive index of sensing medium with Δ*n_s_* = 1 × 10^−4^ and Δ*n_s_* = 1 × 10^−2^. (**e**,**f**) The corresponding sensitivity varying with the incident angle.

We plot sharp resonance arising from coupling between SEP mode and WGM in Figure 5a, and simultaneously plot conventional SEP sensor for comparison in Figure 5b. It is clearly seen that the width of the resonance curve of a conventional SEP sensor is far broader than SEP Fano-type, and a narrower reflectance resonance proves better for sensing detection. We know that the change in the refractive index (*n_s_*) of the sensing medium can lead to a change in reflectance (R) [30]. The reflectance is narrower, and the value of the slope is larger, which means the sensitivity is larger. Then we plot the variation of the reflectance curve for the suggested FR structure (Figure 5c) and SEP sensor (Figure 5d) caused by the change in the refractive index of the sensing medium. Here, we employ the value Δ*n_s_* = 1 × 10^−4^ (corresponding to the proposed structure) and Δ*n_s_* = 1 × 10^−2^ (corresponding to the conventional SEP structure), the different curves ΔR of Figure 5c,d have a maximum value of 0.572 and 0.07. Hence, the sensitivity of the proposed structure is more than three orders higher than the sensitivity in the traditional SEP structure. Figure 5e,f present the sensitivity varying with the incident angle. It is clearly shown that the sensitivity is strongly dependent on the incident angle. Moreover, we all know that FR suffers from significant performance penalties due to the loss of the material, which could not escape the influence of practical application. However, in the actual experiment, the loss of the waveguide layer is induced by different physical origins relying on the approach of the experiment and the manufacturing technique [31].

**Figure 6:**
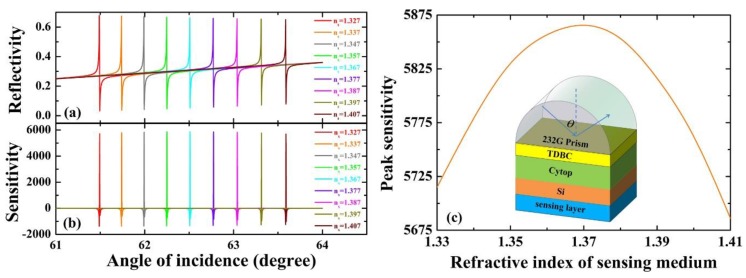
(**a**,**b**) The variations of reflectivity and sensitivity with a different refractive index of sensing medium, λ = 532 nm, d_1_ = 35nm, d_2_ = 300 nm, and d_3_ = 62 nm. (**c**) The peak sensitivity varies with the refractive index of sensing medium.

As in application, the proposed sensor employed the variation of the index of refraction for sensing medium in the final section. We have drawn the effects of the different refractive index of sensing medium on reflectance spectrum and sensitivity in Figure 6a,b, respectively. We know that the peak sensitivity is a function of Δ*n_s_*, and we plot the peak sensitivity in respect to the increase of the refractive index of the sensing medium in Figure 6c. The chart clearly shows that the peak sensitivity increases first and decreases later with the refractive index of sensing medium varying from 1.327 to 1.407; we can get the highest sensitive 5858 RIU^−1^ by selecting an optimal *n_s_*.

## 4. Conclusions

In conclusion, we proposed a multilayer construction that can realize the coupling between SEP mode and WGM. Fano resonance can be used to design an imaging biosensor with the highest sensitivity of 5858 RIU^−1^, which is more than three orders higher than a conventional SEP sensor. According to production cost and degree of manufacturing, exciton material is the best alternative to metal. First of all, we employed the effective index to examine the coupling of the two modes, and then we plotted a three-dimensional diagram of an electric field to explain the origin of sharp resonance. We found that the field strength increased sharply at the interface of waveguide and water in our proposed structure, which can be used in Raman scattering and fluorescence of molecules. Organic excitonic materials are important for achieving novel sensors or devices, and we believe that this novel structure based on SEPs can play an important role in optical sensing technology.

## References

[1] Ha D., Thuy D., Hoa V., Van T., Viet N. (2017). On the theory of three types of polaritons (phonon, exciton, and plasmon polaritons). J. Phys. Conf. Ser..

[2] Barnes W.L., Dereux A., Ebbesen T.W. (2003). Surface plasmon subwavelength optics. Nature.

[3] Kim E.S., Yong M.K., Choi K.C. (2016). Surface-plasmon-assisted nano-lithography with a perfect contact aluminum mask of a hexagonal dot array. Plasmonics.

[4] Homola J., Yee S.S., Gauglitz G. (1999). Surface plasmon resonance sensors: Review. Sens. Actuators B Chem..

[5] Zheng G., Zhang H., Bu L., Gao H., Xu L., Liu Y. (2018). Tunable Fano resonance in mid-infrared waveguide-coupled Otto configuration. Plasmonics.

[6] Zheng G., Chen Y., Bu L., Xu L., Su W. (2016). Waveguide-coupled surface phonon resonance sensors with super-resolution in the mid-infrared region. Opt. Lett..

[7] Takatori K., Okamoto T., Ishibashi K., Micheletto R. (2017). Surface exciton polaritons supported by a J-aggregate-dye/air interface at room temperature. Opt. Lett..

[8] Bonnand C., Bellessa J., Plant J. (2006). Study of strong coupling between surface plasmon and exciton in an organic semiconductor. J. Non-Cryst. Solids.

[9] Pirotta S., Patrini M., Liscidini M., Galli M., Dacarro G., Canazza G., Guizzetti G., Comoretto D., Bajoni D. (2014). Strong coupling between excitons in organic semiconductor and Bloch surface wave. Appl. Phys. Lett..

[10] Ellenbogen T., Steinvurzel P., Crozier K. (2011). Strong coupling between excitons in J-aggregates and waveguide modes in thin polymer films. Appl. Phys. Lett..

[11] Rosenberg J., Shenoi R., Vandervelde T., Krishna S., Painter O. (2009). A multispectral and polarization-selective surface-plasmon resonant midinfrared detector. Appl. Phys. Lett..

[12] Lu H., Liu X., Mao D., Wang G. (2012). Plasmonic nanosensor based on Fano resonance in waveguide-coupled resonators. Opt. Lett..

[13] Miroshnichenko A.E., Flach S.Y., Kivshar S. (2010). Fano resonances in nanoscale structures. Rev. Mod. Phys..

[14] Fano U. (1961). Effects of configuration interaction on intensities and phase shifts. Phys. Rev..

[15] Kaldun A., Ott C., Blattermann A., Laux M., Meyer K., Ding T., Fischer A., Pfeifer T. (2014). Extracting phase and amplitude modifications of laser-coupled Fano resonances. Phys. Rev. Lett..

[16] Zheng C., Jia T., Zhao H., Xia Y., Zhang S., Feng D., Sun Z. (2018). Theoretical study on narrow Fano resonance of nanocrescent for the label-free detection of single molecules and single nanoparticles. RSC Adv..

[17] Zheng C., Liu S., Yang W., Zhu Z. (2016). Coherent control of the Goos-Hanchen shift via Fano interference. J. Appl. Phys..

[18] Hao Q., Juluri B., Zheng Y., Wang B., Chiang I., Jensen L., Crespi V., Eklund P., Huang T. (2010). Effect of intrinsic fano interference on surface-enhanced Raman spectroscopy: Comparison between platinum and gold. J. Phys. Chem. C.

[19] Guo J., Jiang L., Dai X., Xiang Y. (2016). Tunable Fano resonances of a graphene/waveguide hybrid structure at az mid-infrared wavelength. Opt. Express.

[20] Baquedano E., Gonzalez M., Paniagua-Dominguez R., Sanchez-Gil J., Postigo P. (2017). Low-cost and large-size nanoplasmonic sensor based on Fano resonances with fast response and high sensitivity. Opt. Express.

[21] Hayashi S., Nesterenko D., Sekkat Z. (2015). Waveguide-coupled surface plasmon resonance sensor structure: Fano lineshape engineering for ultrahigh-resolution sensing. J. Phys. D Appl. Phys..

[22] Giannini V., Zhang Y., Forcales M., Rivas J.G. (2008). Long-range surface polaritons in ultra-thin films of silicon. Opt. Express.

[23] Maharana P.K., Jha R. (2012). Chalcogenide prism and graphene multilayer based surface plasmon resonance affinity biosensor for high performance. Sens. Actuators B-Chem..

[24] Wu L., Guo J., Xu H., Dai X., Xiang Y. (2016). Ultrasensitive biosensors based on long-range surface plasmon polariton and dielectric waveguide modes. Photonics Res..

[25] Daimon M., Masumura A. (2007). Measurement of the refractive index of distilled water from the near-infrared region to the ultraviolet region. Appl. Opt..

[26] Gupta B., Sharma A.K. (2005). Sensitivity evaluation of a multi-layered surface plasmon resonance-based fiber optic sensor: A theoretical study. Sens. Actuators B-Chem..

[27] Wu L., Chu H.S., Koh W.S., Li E.P. (2010). Highly sensitive graphene biosensors based on surface plasmon resonance. Opt. Express.

[28] Verma R., Gupta B., Jha R. (2011). Sensitivity enhancement of a surface plasmon resonance based biomolecules sensor using graphene and silicon layers. Sens. Actuators B-Chem..

[29] Ruan B., Guo J., Wu L., Zhu J., You Q., Dai X., Xiang Y. (2017). Ultrasensitive terahertz biosensors based on Fano resonance of a graphene/waveguide hybrid structure. Sensors.

[30] Wu L., Guo J., Wang Q., Lu S., Dai X., Xiang Y., Fan D. (2017). Sensitivity enhancement by using few-layer black phosphorus-graphene/TMDCs heterostructure in surface plasmon resonance biochemical sensor. Sens. Actuators B-Chem..

[31] Song M., Yu H., Wang C., Yao N., Pu M., Luo J., Zhang Z., Luo X. (2015). Sharp Fano resonance induced by a single layer of nanorods with perturbed periodicity. Opt. Express.

